# Candidemia due to *Candida lambica* in a neutropenic oncology patient: A rare case report

**DOI:** 10.1016/j.mmcr.2025.100720

**Published:** 2025-07-23

**Authors:** Sanam Nami, Mortaza Raeisi, Zahra Ramezanalipour, Parviz Hassanpour

**Affiliations:** a Department of Parasitology and Mycology, Faculty of Medicine, Tabriz University of Medical Sciences, Tabriz, Iran; b Hematology and Oncology Research Center, Tabriz University of Medical Sciences, Tabriz, Iran; c Department of Parasitology and Mycology, School of Public Health, Tehran University of Medical Sciences, Tehran, Iran

**Keywords:** *Candida lambica*, Antifungal susceptibility, Acute myeloid leukemia

## Abstract

We report a rare case of *Candida lambica* candidemia in a 56-year-old male with acute myeloid leukemia undergoing intensive chemotherapy. Despite fluconazole prophylaxis, the patient developed persistent febrile neutropenia. Blood cultures grew yeast, later identified as *C. lambica* via ITS rDNA sequencing. Antifungal susceptibility testing revealed sensitivity to voriconazole and other antifungals resulting in clinical improvement following targeted therapy. This case highlights the growing clinical relevance of rare *non-albicans candida* species in immunocompromised hosts, the limitations of conventional diagnostics, and the importance of molecular tools and antifungal susceptibility testing in guiding effective treatment.

## 1 Introduction

Bloodstream Infections (BSIs) pose significant challenges for healthcare systems, exhibiting specifically high mortality rates when fungal pathogens are involved. A specific form of BSI is Candidemia, which is caused by the presence of different *Candida* species in the bloodstream. [1,2]. Among *Candida* species, *C. albicans* remains the most common cause of candidemia. In recent years, the increase in cases of candidemia with *non-albicans candida*(NAC) has raised concerns about the diagnosis, treatment, and control of these infections [3]. Key risk factors for candidemia include extended hospital stays, the use of central venous catheters(CVC), prolonged antibiotic therapy, immunodeficiency, major surgical procedures, and parenteral nutrition. Early diagnosis through blood culture and molecular methods, combined with the selection of appropriate antifungal therapy based on the identified species, is critical for optimizing patient prognosis [4]. Given the complexities associated with the targeted identification of *Candida* species and the drug resistance exhibited by some strains, it is essential to deepen our understanding of the species and investigate their epidemiological characteristics, risk factors, diagnostic methods, and treatment strategies. [4,5]. Multiple investigations worldwide, including those from Iran, have increasingly reported candidemia caused by NAC, indicating a shift in the epidemiological pattern of invasive candidiasis. The present report contributes to this growing body of evidence by documenting an additional case caused by a rare species, *C. lambica*. This yeast, with the teleomorph *Pichia fermentans*, was initially described as *Mycoderma lambica* by Linder and Genoud in 1913 and was long mistakenly identified as *Candida krusei* (teleomorph: *Issatchenkia orientalis*) due to morphological similarities [5]. Its isolation underscores the importance of accurate species identification in immunocompromised populations, where NAC may be emerging pathogens with distinct antifungal resistance profiles and clinical implications. Herein, we report the first documented case of candidemia caused by *C. lambica* in Iran and provide a comprehensive review of the literature on *C. lambica* fungemia.

## 2 Case presentation

**Fig. 1:**
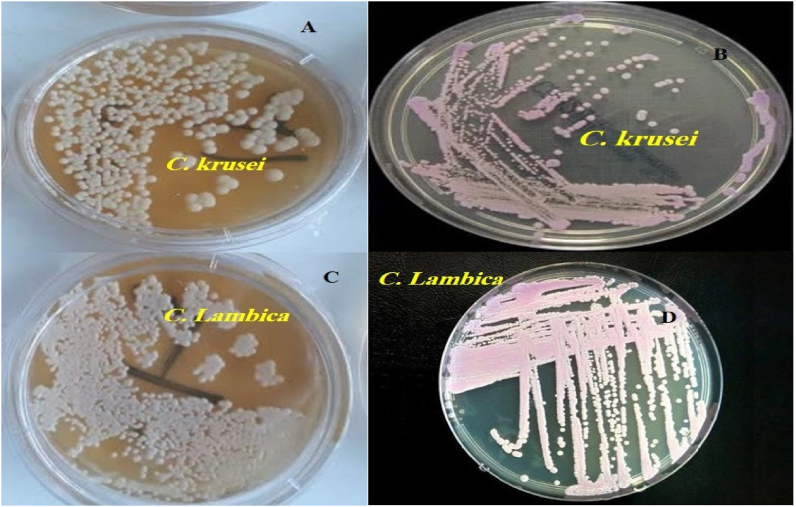
A and C: On SDA medium, both *C. krusei* and *C. lambica* produce cream-colored colonies with similar appearance. B: On CHROMagar *Candida* medium *C. krusei* forms dry, pink to purple colonies and D: *C. lambica* produces colonies with a paler and less distinct purple hue. This similarity in colony morphology highlights the potential for misidentification when relying solely on conventional mycological methods.

Our case was a 56-year-old male patient with a confirmed diagnosis of acute myeloid leukemia (AML) who was admitted to the oncology Shahid Ghazi Tabatabaei Hospital in Tabriz, Iran [6]. The patient had been hospitalized for 28 days and received chemotherapy throughout this period. To address prolonged neutropenia following AML treatment, ciprofloxacin (500 mg daily) was prescribed as prophylaxis against bacterial infections, along with oral fluconazole (FLU) (200 mg daily) to prevent fungal infections. Despite receiving prophylactic treatment, the patient's condition deteriorated progressively. On day 0 (Day 29 of hospitalization), the patient developed initial symptoms, presenting with a high-grade fever accompanied by rigors, profuse diaphoresis, and tachycardia. Physical examination revealed hypotension (BP 90/60 mmHg) and reduced oxygen saturation (SpO_2_ 88 %). At this point, systemic infection (sepsis) was suspected, and the patient was promptly transferred to the intensive care unit (ICU). Laboratory parameters showed a white blood cell (WBC) count of 1.7 × 10 ^3^/μl (i.e., leukopenia), neutrophil count of 350 cell/μl (i.e., Neutropenia), hemoglobin (HB) level of 6.2 g/dl, platelet count of 12 × 10 ^3^/μl, c-reactive protein (C-RP) level of 74 mg/L, and erythrocyte sedimentation rate (ESR) level of 35 mm/hr. On day 1, 10 ml of venous blood samples were aseptically collected from the patient during the fever. The blood samples were inoculated into blood culture bottles (BACTEC Myco/F Lytic culture vials) and processed using the BACTEC 9120 automated blood culture system (Becton Dickinson Microbiology Systems, Maryland, DE, USA) during a 5-day incubation period. Blood samples were then inoculated into brain-heart infusion (BHI) bottles (Baharafshan, Iran) and incubated for 7 days at 37 °C. Initial blood cultures showed no bacterial growth but were positive for yeast-like fungi. Yeast growth was first detected in the BACTEC system on Day 5, while BHI broth was used simultaneously but yielded confirmatory growth on day 7. Positive blood cultures were subcultured on Sabouraud Dextrose Agar (SDA) and CHROMagar™ Candida medium (CHROMagar Microbiology, Paris, France). On day 9, colonies on SDA at 25 °C appeared white to cream-yellow, dry, and slightly wrinkled. On CHROMagar, they exhibited a faint pink coloration (Fig. 1). Microscopic examination revealed yeast cells displaying active budding with limited pseudohyphae formation, consistent with the morphological features characteristic of the *Candida* genus. Following the identification of the *Candida* species, antifungal prophylaxis with FLU was discontinued, and treatment with voriconazole (VRC) was initiated on day 9. Pure colonies were transferred to Eppendorf tubes containing sterile water, and identification at the species level was performed using DNA sequencing.

**Fig. 2:**
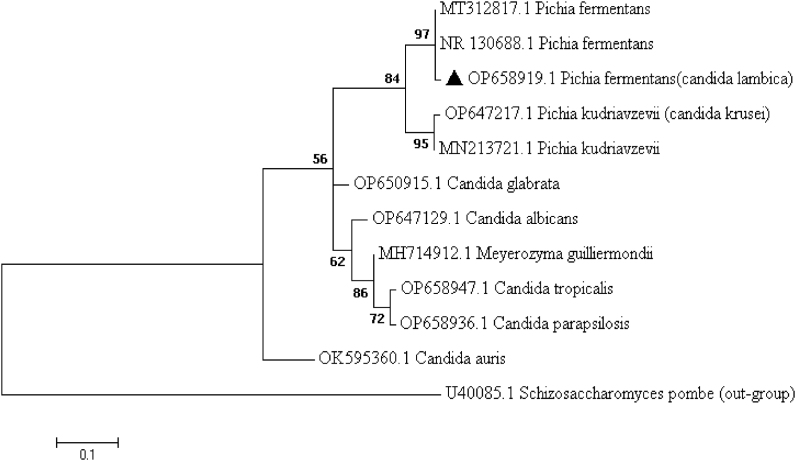
Phylogenetic tree based on the ITS1 and ITS4 gene regions of Candida species using the two-parameter Kimura model in the maximum likelihood algorithm with 1000 bootstrap resampling. Our recorded sequences in this study are highlighted.

On day 11, genomic DNA was extracted from two- and three-day-old cultures grown on SDA using the boiling method [7]. The internal transcribed spacer (ITS) was amplified and sequenced using primers ITS1-5.8S-ITS2 rDNA region via ITS1/ITS4 primer pairs (ITS1: 5′- TCC GTA GGT GAA CCT GCG G-3′; ITS4: 5′-TCC TCC GCT TAT TGA TAT GC-3′) (Sinaclon, Iran). The results of Sanger sequencing were analyzed and edited via Sequencher 4.7 and MEGA 11 software. The sequences were aligned using the Basic Local Alignment Search Tool (BLAST) and compared against fungal sequences available in the (Gen Bank) database to assess their similarities. The generated ITS sequence was ultimately deposited in GenBank under accession number OP658919.1 (*C. lambica*). To confirm the genetic relationship of the identified isolate with other *Candida* species, a phylogenetic tree was generated by MEGA 11 software, and the cladistic analysis demonstrated a sister relationship between the *Pichia kudriavzevii* (*C. krusei*) clade and the *Pichia fermentans* (*C. lambica*) clade (Fig. 2).

Simultaneously**,** antifungal susceptibility testing (AFST) was conducted following the methodology described in the CLSI documents M27-A3 [8]. The antifungal agents used in this study included amphotericin B (AMB), fluconazole (FLU), voriconazole (VRC), itraconazole (ITR), and caspofungin (CSP), all obtained from Merck (Whitehouse Station, NJ, USA). Exhibiting minimum inhibitory concentrations (MIC) values of 1 mg/L for AMB, 4 mg/L for FLU,0.5 mg/L for ITR, 1 mg/L for VRC, and 0.12 mg/L for CSP. The isolate demonstrated susceptibility to all antifungals. Due to the rarity of *C. lambica*, clinical breakpoints (CBPs) and epidemiological cutoff values (ECVs) have not been established for this species by CLSI. Therefore, the obtained MIC values are reported descriptively and have been compared with the CBPs or ECVs of more common *Candida* species [9]. However, any clinical interpretation of these results should be made with caution, given the lack of species-specific reference standards. Following the identification of the fungal pathogen and its antifungal susceptibility profile, prophylactic antifungal therapy was discontinued. On day 9, in line with hospital protocol and given the patient's ability to tolerate oral medications, targeted treatment was initiated with the removal of the CVC and the administration of oral VRC with a loading dose of 400 mg every 12 hours for the first two days, followed by a maintenance dose of 200 mg every 12 hours. No replacement catheter was inserted, and peripheral IV access was maintained throughout treatment. During the course of treatment, blood cultures were monitored regularly to assess the therapeutic response, and they turned negative on day 14, which was five days after the initiation of VRC. Treatment with antifungal agents continued for at least two weeks after blood cultures became negative and neutropenia had resolved. By the end of the treatment period, the patient showed partial clinical improvement. The treatment period, spanning from the initial appearance of clinical symptoms to full recovery, lasted approximately one month.

## 3 Discussion

Bloodstream Infections represent a significant clinical challenge in immunocompromised patients, particularly those affected by hematologic malignancies, with AML patients being among the most vulnerable [6]. In this case, we describe candidemia caused by *C. lambica* in a neutropenic AML patient receiving induction chemotherapy despite FLU prophylaxis. A recurring theme across the literature is the difficulty in accurately identifying *C. lambica* to the species level using conventional methods, such as CHROMagar™ morphology and microscopic examination. In our case, while preliminary morphology suggested a *Candida* species, confirmation required molecular characterization via Sanger sequencing of the ITS1-5.8S-ITS2 rDNA region (GenBank accession no. OP658919.1.). Phylogenetic analysis revealed a close relationship to *Pichia kudriavzevii (C. krusei)*, a species known for intrinsic FLU resistance, emphasizing the importance of molecular tools to avoid misidentification and inappropriate treatment. This approach parallels the diagnostic pathway described by Vervaeke et al. [10], in which a *C. lambica* isolate from an intravenous drug user was initially misidentified as *C. krusei* through CHROMagar *Candida* medium based on its colony morphology but subsequently confirmed as *C. lambica* by ITS sequencing. Another study further underscores the role of advanced diagnostics, noting that rare *Candida* species, such as *C. lambica,* were increasingly identified only after utilizing matrix-assisted laser desorption/ionization time-of-flight mass spectrometry MALDI-TOF MS [11].

The immunosuppressive profile in our patient, including chemotherapy-induced neutropenia, anemia, and thrombocytopenia, is a recognized risk factor for invasive fungal disease. Krüger et al. [12] supported this notion by showing that prolonged neutropenia and mucosal barrier damage were common among patients with fungal infections; notably, one case with *C. lambica* in that study survived following liposomal AMB therapy. Likewise, from a 12-year retrospective study by Noni et al. [13] in a children's Hospital, *C. lambica* (teleomorph *Pichia fermentans*) was recovered from the blood of an ICU infant and showed susceptibility to AMB, flucytosine, and VRC. In our case, an immunocompromised adult patient developed *C. lambica* candidemia during a critical illness phase, with the isolate demonstrating similar antifungal susceptibility. These cases collectively underscore the emerging pathogenic role of *C. lambica* in high-risk hosts and its consistent in vitro responsiveness to azole and polyene agents. Trowbridge et al. [14] also reported a case of polyarthritis caused by *C. lambica* in a chronic alcoholic, potentially reflecting how reduced neutrophil counts in such individuals compromise innate immunity and increase susceptibility to opportunistic fungal infections. Hematogenous spread to multiple joints in their case mirrors the systemic dissemination observed in ours, underscoring the organism's ability to invade sterile sites when host defenses are impaired.

The clinical presence of this yeast is also explored by Aydin et al. [15], in a study that reports *P. fermentans* isolates from skin lesions in healthcare workers linked to extended use of personal protective equipment (PPE) during the COVID-19 pandemic. Our case also experienced hospital-based risk factors, such as prolonged hospitalization and catheterization, which raise questions about the persistence of this organism on surfaces, healthcare personnel, or even water systems, further supporting infection control interventions like meticulous catheter maintenance and environmental monitoring.

Our isolate showed in vitro susceptibility to AMB, FLU, VRC, ITR, and CSP, which was in line with the results of Vervaeke et al. [10], who achieved susceptible MIC values of 0.125, 0.064 and 2 μg/ml for AMB, ITC, and flucytosine, respectively, and a resistant MIC value of >64 for FLU. Also, this finding resonates with the limited cases of *C. lambica*, which demonstrate variable susceptibility to various antifungals [[16], [17], [18]]. Collectively, this evidence supports the continued efficacy of non-azole antifungals as viable treatment options for *C. lambica*. Although guided by AFST results and Infectious Diseases Society of America (IDSA) protocols, oral VRC was administered, resulting in a favorable clinical outcome and underscoring the importance of susceptibility-directed therapy in managing such complex infections [19,20].

Despite FLU prophylaxis, our patient developed breakthrough fungemia, suggesting probable subtherapeutic exposure, acquired resistance, or biofilm-related protection via the CVC. Kaur et al. [11] support this concern, reporting rising MICs for AMB and consistent FLU resistance across *Candida* species, further justifying the use of broader-spectrum azoles like VRC in high-risk settings. Although Aydin et al. [15] primarily investigated superficial infections linked to PPE use, they observed notable levels of resistance among *Candida* and *Pichia* isolates (47.6 % to FLU and 76.2 % to VRC), highlighting considerable variability in azole susceptibility within these species. In our case, VRC was selected based on MIC data and appropriately dosed; clinical improvement was partial, aligning with Krüger et al. [19], who found only *C. lambica* responded to liposomal AMB among seven cases, while *C. krusei* and *C. glabrata* infections were often fatal. A subsequent study by the same group confirmed liposomal AMB's tolerability in stem cell transplant recipients and suggested *C. lambica* may be less virulent and more treatable than other NAC.

## 4 Limitations of the study

This case report has several limitations. The absence of advanced diagnostic tools like MALDI-TOF MS and molecular methods such as multilocus sequence typing (MLST) or whole-genome sequencing (WGS) limited the depth and confirmation of fungal identification. Additionally, the lack of broader epidemiological data prevented assessment of possible nosocomial transmission. Finally, resistance mechanisms were not investigated, leaving the cause of breakthrough candidemia despite FLU prophylaxis unclear.

## 5 Strengths of the study

This is among the first reported cases of *C. lambica* BSI in an AML patient in Iran and possibly the Middle East. It adds to the scarce data on rare Candida species in immunocompromised hosts. A key strength was accurate species identification via ITS sequencing, overcoming limitations of phenotypic methods and minimizing misidentification with species like *C. krusei*. Phylogenetic analysis further validated the findings.

## CRediT authorship contribution statement

**Sanam Nami:** Writing – review & editing, Writing – original draft, Conceptualization. **Mortaza Raeisi:** Writing – review & editing, Investigation. **Zahra Ramezanalipour:** Writing – review & editing, Investigation. **Parviz Hassanpour:** Writing – review & editing, Investigation, Conceptualization, preparation of the final version.

## Consent

Written informed consent was obtained from the patients for publication of this case series and accompanying images. A copy of the written consent is available for review by the Editor-in-Chief of this journal on request.

## Funding

This study was financially supported by the Infectious and Tropical Diseases Research Center, Tabriz University of Medical Sciences, Iran, (Grant No: IR.TBZMED.REC.1399.1157).

## Declaration of competing interest

The authors declare no conflicts of interest or personal relationships that could have appeared to influence the work reported in this paper.
