# Supplementary material for: Candidemia due to Candida lambica in a neutropenic oncology patient: A rare case report
Source: Med Mycol Case Rep. 2025 Jul 23;49:100720. doi: 10.1016/j.mmcr.2025.100720 (PMC12311543; doi:10.1016/j.mmcr.2025.100720)
Supplement: Multimedia component 1 [file mmc1.pdf]

Medical Mycology Case Reports
ETHICAL FORM

Manuscript number (if applicable):

Author name:
Sanam Nami, Mortaza Raeisi, Zahra Ramezanalipour, Parviz Hassanpour

Medical Mycology Case Reports requires full disclosure of all sources of funding and potential conflicts of interest. The journal also requires a declaration that the author(s) have obtained written and signed consent to publish the case report from the patient or legal guardian(s).

If you have nothing to declare in any of these categories then this should be stated.

Funding Source

All sources of funding should be acknowledged and you should declare any extra funding you have received for academic research of this work. If there are none state ‘there are none’.

Please state any sources of funding for your research

This study was financially supported by the Tabriz University of Medical Sciences Iran, (Grant No: IR.TBZMED.REC.1399.1157)

Conflict of Interest

Please declare any financial or personal interests that might be potentially viewed to influence the work presented. Interests could include consultancies, honoraria, patent ownership or other. If there are none state ‘there are none’.

Please state any competing interests

The authors declare no conflicts of interest or personal relationships that could have appeared to influence the work reported in this paper.

Consent

Please declare that you have obtained written and signed consent to publish the case report from the patient or legal guardian(s).

Please state that consent has been obtained from the patient or legal guardian(s)

Written informed consent was obtained from the patient or legal guardian(s) for publication of this case report and accompanying images. A copy of the written consent is available for review by the Editor-in-Chief of this journal on request.

As corresponding author, I hereby declare that I sign this document on behalf of all the authors of the above mentioned manuscript.

Signature (a scanned signature is acceptable)

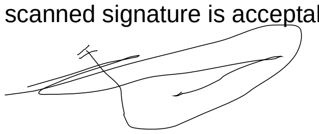

Print name

parviz Hassanpour

_______________________________________ ___________________________
